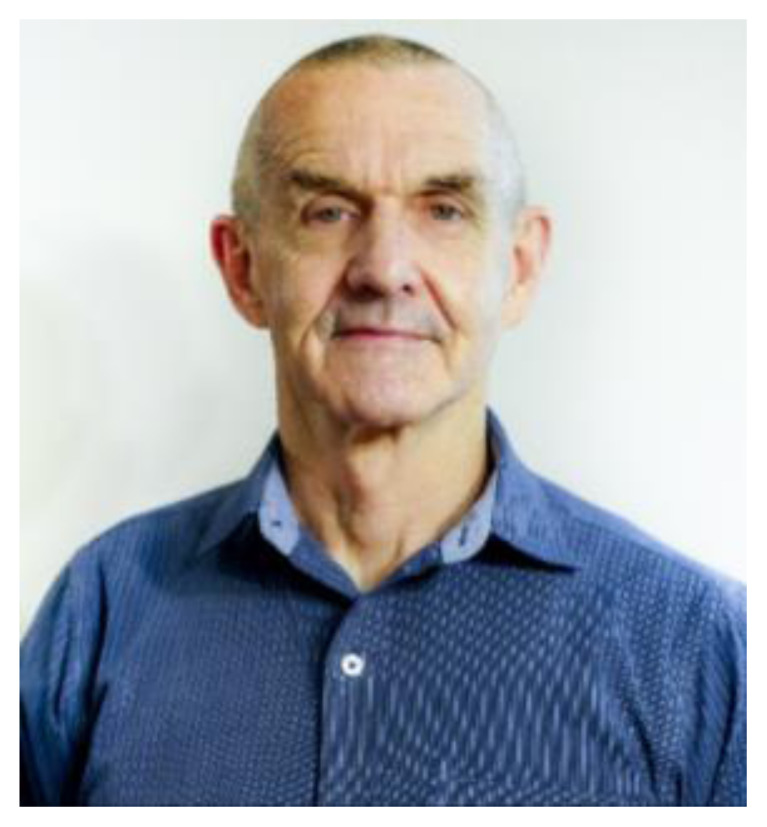# South African Journal of Sports Medicine: 2020 what lies ahead?

**DOI:** 10.17159/2078-516X/2020/v32i1a7909

**Published:** 2020-01-01

**Authors:** Mike Lambert

**Affiliations:** Editor-in-chief

The start of 2020 provides an opportunity to reflect on 2019 to determine how the South African Journal of Sports Medicine developed. This should be aligned to the journal’s goal to promote research associated with sport and exercise medicine, in particular, research which addresses questions relevant to sport, exercise, nutrition and health in South Africa.

In 2019, the journal published eight original research papers, two commentaries, one invited review, one case study, two reports and two conference proceedings. Although the number of outputs is relatively low, on closer inspection, the majority of outputs have direct relevance to the journal’s target audience in South Africa. A journal has to continually find the balance between outputs which are relevant to the target audience and ensuring that these papers are of a high standard. This is a difficult balance to achieve because most authors with high quality work, even with a local relevance, will try and get their work published in high impact international journals. While the South African Journal of Sports Medicine could publish lower quality research papers to keep the number of outputs high, we believe this is not the correct decision. Instead our strategic decision is to maintain the quality of the outputs at the expense of quantity. Less than 50% of the papers submitted for review are accepted. This means that the journal’s growth, defined by the quantity of outputs, will be slower than desired. Growth defined by output quality is more likely to place the South African Journal of Sports Medicine on the trajectory that will result in it getting International Scientific Indexing (ISI) accreditation sooner. This is the desired long-term goal which will enable the journal to become more dominant in this subject field, thereby attracting a greater number of good quality papers. The South African Journal of Sports Medicine is already accredited by South Africa’s Department of Higher Education and Training which enables researchers at South African institutions to get a research subsidy following publication in the journal. The papers published in the journal are also listed by several electronic databases, a full listing of which can be found on our website.

The quality of any journal depends on the quality of the reviewers. In most cases, the reviewers have done their job well and provided good quality, thoughtful reviews. This not only assists the authors of the paper to improve their publications, but also raises the standard of the journal. The work by the reviewers is done anonymously and without pay. For this they are thanked in writing by the journal. We have to compete with other journals for reviewers. A mid- to high-tier quality scientist may receive several requests a week to review papers from other journals, so we appreciate the reviewers who agree to assist us. We are planning to have a training workshop during the year to train aspiring reviewers and sharpen the skills of those reviewers who have already some experience.

The field of exercise and sports medicine continues to evolve. Sometimes the questions with regard to the topics of the subject field are answered and at times the topic loses relevance. Concussion during sport is an area within sports medicine which has attracted scientists from different disciplines. Later this year the 6^th^ International Conference on Concussion in Sport will be held in Paris. This is a prestigious conference which defines the current knowledge on sports-related concussion and research for the future. The conference on sports-related concussion started in 2001 and meets every four years. It is supported by the following: International Olympic Committee, Fédération Internationale de Football Association (FIFA), International Federation for Equestrian Sports, World Rugby and the International Ice Hockey Federation. The Sport Concussion Assessment Tool (SCAT) was developed from the conference held in 2005. This tool has become entrenched in sports medicine and is used around the world in a variety of sports. The tool has undergone several revisions to make it a valid and reliable tool and is likely to be tweaked further at this conference. A Scientific Committee coordinates the programme to ensure the knowledge in the area advances. The expert panel at the conference will develop a further Consensus Statement on Concussion in Sport, building on the previous consensus statements which have been published after each conference. ^[1,2]^ This document will define the management of athletes following a head injury during sport, using the most up-to-date evidence. The progress the scientists participating in the concussion movement have made shows how a focused, well-directed approach can transform concussion knowledge and, in particular, the management of athletes following a head injury. This is a good model for scientists working in other focused areas.

**Figure:**